# Memory enhancement by multidomain group cognitive training in patients with Parkinson’s disease and mild cognitive impairment: long-term effects of a multicenter randomized controlled trial

**DOI:** 10.1007/s00415-021-10568-9

**Published:** 2021-04-27

**Authors:** Nele Schmidt, Inken Tödt, Daniela Berg, Christian Schlenstedt, Ann-Kristin Folkerts, Anja Ophey, Karina Dimenshteyn, Saskia Elben, Lars Wojtecki, Inga Liepelt-Scarfone, Claudia Schulte, Patricia Sulzer, Carsten Eggers, Elke Kalbe, Karsten Witt

**Affiliations:** 1 Department of Neurology, University Oldenburg, Steinweg 13-17, 26122 Oldenburg, Germany; 2 Department of Neurology, University Hospital Schleswig-Holstein, Christian-Albrechts-University, Kiel, Germany; 3 Medical Psychology, Neuropsychology and Gender Studies, Center for Neuropsychological Diagnostics and Interventions (CeNDI), Faculty of Medicine and University Hospital Cologne, University of Cologne, Cologne, Germany; 4 Department of Neurology, Center for Movement Disorders and Neuromodulation and Institute of Clinical Neuroscience and Medical Psychology, Medical Faculty, Heinrich-Heine-University Duesseldorf, Duesseldorf, Germany; 5 Department of Neurodegenerative Diseases, German Center for Neurodegenerative Diseases (DZNE) and Hertie Institute for Clinical Brain Research, University of Tuebingen, Tuebingen, Germany; 6 IB Hochschule Für Gesundheit Und Soziales, Stuttgart, Germany; 7 Department of Neurology, University Hospital of Marburg, Center for Mind, Brain and Behavior (CMBB), Universities Marburg and Giessen, Marburg, Germany; 8 Department of Neurology, Faculty of Medicine and University Hospital Cologne, University of Cologne, Cologne, Germany; 9 Research Center Neurosensory Science, Carl von Ossietzky University Oldenburg, Oldenburg, Germany

**Keywords:** Parkinson’s disease, Mild cognitive impairment, Cognition, Cognitive training, Non-pharmacological intervention, Long-term effects

## Abstract

**Background:**

Meta-analyses indicate positive effects of cognitive training (CT) in patients with Parkinson’s disease (PD), however, most previous studies had small sample sizes and did not evaluate long-term follow-up. Therefore, a multicenter randomized controlled, single-blinded trial (Train-ParC study) was conducted to examine CT effects in PD patients with mild cognitive impairment (PD-MCI). Immediately after CT, an enhancement of executive functions was demonstrated. Here, we present the long-term results 6 and 12 months after CT.

**Methods:**

At baseline, 64 PD-MCI patients were randomized to a multidomain CT group (*n* = 33) or to a low-intensity physical activity training control group (PT) (*n *= 31). Both interventions included 90 min training sessions twice a week for 6 weeks. 54 patients completed the 6 months (CT: *n *= 28, PT: *n *= 26) and 49 patients the 12 months follow-up assessment (CT: *n *= 25, PT: *n *= 24). Primary study outcomes were memory and executive functioning composite scores. Mixed repeated measures ANOVAs, post-hoc *t* tests and multiple regression analyses were conducted.

**Results:**

We found a significant time x group interaction effect for the memory composite score (*p *= 0.006, *η*^2^ = 0.214), but not for the executive composite score (*p *= 0.967, *η*^2^ = 0.002). Post-hoc *t* tests revealed significant verbal and nonverbal memory improvements from pre-intervention to 6 months, but not to 12 months follow-up assessment in the CT group. No significant predictors were found for predicting memory improvement after CT.

**Conclusions:**

This study provides Class 1 evidence that multidomain CT enhances memory functioning in PD-MCI after 6 months but not after 12 months, whereas executive functioning did not change in the long-term.

**Clinical trial registration:**

German Clinical Trials Register (ID: DRKS00010186), 21.3.2016 (The study registration is outlined as retrospective due to an administrative delay. The first patient was enrolled three months after the registration process was started. A formal confirmation of this process from the German Clinical Trials Register can be obtained from the authors.)

**Supplementary Information:**

The online version contains supplementary material available at 10.1007/s00415-021-10568-9.

## Introduction

Cognitive impairment is a common non-motor symptom in idiopathic Parkinson’s disease (PD) with a prevalence of approximately 40% [1]. Since cognitive deficits have a negative impact on patients’ quality of life (QoL) [2], increase mortality [3] and so far only limited pharmacological treatment options are available [4, 5], there is a need for research in non-pharmacological interventions. Two meta-analyses showed positive effects of cognitive training (CT) in PD patients regarding executive functioning, working memory, memory, processing speed, or attention with small to medium effect sizes [6, 7]. A review on non-pharmacological management of cognitive impairment in PD reported level B evidence for improving or maintaining memory, attention and working memory performance after CT [8], while another recent review on CT in PD patients with mild cognitive impairment (PD-MCI) and PD dementia did not find clear evidence that CT improves cognitive functioning [9]. However, the authors emphasize the low level of certainty due to small sample sizes, the heterogeneous study population concerning varying degrees of cognitive impairment, and the lack of studies reporting on long-term effectiveness. Moreover, little research has been done in the past to identify predictors of CT responsiveness in PD patients. Few previous studies systematically investigated a variety of sociodemographic, clinical, genetic, and neuropsychological factors [10–14], however, inconsistent results were reported for most predictors.

Our recently published multicenter randomized controlled trial (RCT) that is directly linked to the present study analyzed the short-term results of CT in PD-MCI patients compared to an active physical training control group (PT) [15]. In the CT group, an enhancement of executive functions (especially verbal fluency) and self-reported physical activity could be demonstrated while working memory improved in the PT group. In the memory domain, however, no significant training gains were found. Baseline cognitive levels, education, disease progression, and Apolipoprotein E4 (ApoE4) state were significant predictors for training responsiveness, indicating that vulnerable patients benefit the most from CT. Also, it could be shown that CT is feasible and safe for PD-MCI patients. Here, we report the long-term results of the study at 6 and 12 months follow-up assessments after CT. We aimed (1) to examine the long-term efficacy of CT regarding memory and executive functioning as well as further secondary cognitive and non-cognitive outcome parameters in PD-MCI, and (2) to identify predictors for training responsiveness at these follow-up time points.

## Methods

### Study design

The study is registered in the German Clinical Trials Register (ID: DRKS00010186) and was approved by the local ethic committees of all participating centers. All patients gave their informed consent in written form. Data were collected in four German university hospitals (Cologne, Duesseldorf, Tuebingen, Kiel) between July 2016 and May 2018. A priori sample size calculation focused on short-term training effects showed that an overall sample size of *n *= 80 at baseline is necessary to achieve 80% power at a significance level at *p *= 0.05 when considering a 10–15% drop-out rate. The participants were randomized to the CT or PT group and the persons who carried out the outcome investigations were blinded for intervention type. The patients were assessed pre- and post-intervention as well as 6 and 12 months after intervention, each assessment within a time frame of 4 weeks based on the first or last session of the intervention. All intervention sessions and diagnostic examinations were performed under regular antiparkinsonian medication. Data were entered in a secured online database system in pseudonymized form. Data monitoring was carried out by two members of another study site. For a detailed reporting on study design, randomization procedure and data management following the CONSORT statement, please see Kalbe et al. [15].

### Patients

All patients were diagnosed with PD according to the UK Brain Bank criteria [16] and PD-MCI according to the Movement Disorders Society task force Level-II criteria [17] requiring impairment in at least two cognitive tests (operationalized as at least one standard deviation below the mean normative score). Further inclusion criteria were age between 50 and 80 years and a PD duration of at least three years with a stable medication within four weeks before screening procedure as well as subjective cognitive impairment as diagnosed using the Subjective Cognitive Impairment questionnaire [18] and/or objective cognitive impairment in the Montreal Cognitive Assessment [19] (cut-off < 26 points). Exclusion criteria were a clinical PD dementia diagnosis according to the criteria of Emre et al. [20], impaired activities of daily living (ADL) according to the Pill Questionnaire [21] (impact on daily living is supposed when the patient cannot describe his or her regular medication and in case of doubt a caregiver confirms that he or she is no longer able to take the pills safely and reliably without supervision), and severe depression measured with the Beck Depression Inventory II [22] (cut-off ≥ 20 points, range 0–63 points, higher scores indicate more severe signs and symptoms of depression). In an anamnestic interview, the following exclusion criteria were evaluated: suicide tendency, severe comorbidities, severe fatigue, prominent impulse control disorder or dopamine dysregulation syndrome, acute psychosis or psychotic episode in the last six months, dementia medication, participation in other treatment studies within the last two months, pregnancy, or deep brain stimulation.

### Interventions

As CT, the NEUROvitalis program [23] was conducted. In this standardized training program, executive functions, memory, attention, and visuocognition are trained by group tasks, activity games, individual exercises, and homework. Furthermore, it contains psychoeducative elements, e.g. explaining cognitive functions and strategies to enhance these functions. Two sessions of the original version of the program were modified in consideration of the characteristic cognitive profile of PD patients. More precisely, two memory sessions were replaced by sessions focusing on executive functions and visuocognition. The modified program was recently published as NEUROvitalis Parkinson [24]. The PT group received a low-intensity physical activity program which aimed to improve motor function but not cognition. Each session included warm-up exercises, specific exercises focusing on stretching, flexibility, loosening up, or relaxation, psychoeducation, and homework. Both training programs were conducted in groups with three to five patients and included two 90 min sessions a week over a total of six weeks. As part of CT and PT, patients were encouraged to stimulate themselves cognitively and physically after the end of the training phase, but no new training sessions or exercises were conducted until the follow-up assessments. For further details of the study interventions, we refer to Supplementary Table 1 in the article by Kalbe et al. [15].

**Table 1:** Sociodemographic and clinical baseline characteristics of the PD-MCI subgroups that are included in the 6 respective 12 months follow-up analyses

	6 months follow-up			12 months follow-up		
	Cognitive training (*n* = 28)	Physical training (*n* = 26)	*p*	Cognitive training (*n* = 25)	Physical training (*n* = 24)	*p*
Age (years)	67.18 ± 7.01	67.50 ± 8.71	0.881^a^	67.04 ± 6.63	67.08 ± 8.85	0.985^a^
Sex						
Male (%)	21 (75%)	15 (57.7%)	0.250^c^	19 (76%)	14 (58.3%)	0.232^c^
Female (%)	7 (25%)	11 (42.3%)		6 (24%)	10 (41.7%)	
Years of education	13.43 ± 3.84	13.96 ± 3.33	0.868^b^	13.20 ± 3.74	13.92 ± 3.20	0.769^b^
Age of PD symptom onset (years)	58.11 ± 8.61	59.35 ± 9.04	0.613^a^	57.92 ± 7.60	59.25 ± 9.36	0.591^a^
Age at PD diagnosis (years)	59.29 ± 8.87	59.96 ± 9.11	0.784^a^	59.12 ± 8.07	59.88 ± 9.15	0.764^a^
PD duration (months)	93.07 ± 66.32	89.54 ± 44.88	0.917^b^	93.52 ± 68.0	85.67 ± 44.53	0.772^b^
Hoehn and Yahr stage						
1 (%)	2 (7.1)	6 (23.1)	0.113^d^	2 (8.0)	5 (20.8)	0.273^d^
2 (%)	16 (57.1)	17 (65.4)		15 (60.0)	16 (66.7)	
3 (%)	9 (32.1)	3 (11.5)		7 (28.0)	3 (12.5)	
4 (%)	1 (3.6)	0		1 (4.0)	0	
5 (%)	0	0		0	0	
UPDRS-III	25.43 ± 13.26	25.08 ± 12.80	0.931^b^	25.04 ± 12.34	26.21 ± 12.68	0.681^b^
LEDD	890.80 ± 519.80	739.58 ± 411.85	0.411^b^	935.22 ± 530.77	739.92 ± 425.73	0.250^b^
ApoE4 carriers	5 (17.9%)	3 (11.5%)	0.711^d^	4 (16%)	3 (12.5%)	1.000^d^
BDI-II	8.43 ± 5.65	7.28 ± 4.11	0.616^b^	8.28 ± 5.76	7.57 ± 4.17	0.868^b^
MoCA	25.0 ± 2.22	24.23 ± 3.15	0.340^b^	25.08 ± 2.08	24.13 ± 3.26	0.266^b^

Results are given in mean ± standard deviation

*BDI* Beck Depression Inventory, *LEDD* Levodopa equivalent daily dose, *MoCA* Montreal Cognitive Assessment, *PD* Parkinson’s Disease, *UPDRS* Unified Parkinson’s Disease Rating Scale

^a^*t* test

^b^Mann–Whitney *U* test

^c^*χ*^2^ test

^d^Fisher’s exact test

### Outcomes

Primary study outcomes were (i) a composite score for memory and (ii) a composite score for executive functions, both defined as averaged z-scores of the respective cognitive test parameters. Secondary outcomes were composite scores for attention, working memory, visuocognition, and language, as well as single test results for ADL, self-reported physical activity, depression, QoL, self-experienced attention deficits, motor impairment, and freezing of gait. The Diagnostic Tests used were the following:

- Memory: California Verbal Learning Test (CVLT) [25]—total score trials 1–5 and long delay free recall II, Rey-Osterrieth Complex Figure Test (ROCFT) [26]—delayed recall.
- Executive functions: Regensburger word fluency tests [27]—phonemic and semantic word fluency, modified card sorting test [28]—categories completed, Behavioural Assessment of the Dysexecutive Syndrome [29]—Key Search test.
- Attention: d2-R [30]—errors and concentration performance.
- Working memory: Wechsler Adult Intelligence Scale III [31]—letter-number sequencing and digit span backwards.
- Visuocognition: ROCFT—copy, Benton Judgment of Line Orientation [32].
- Language: Consortium to Establish a Registry for Alzheimer's Disease [33]—Boston Naming Test, Aphasia Check List [34]—speech comprehension.
- ADL: Bayer Activities of Daily Living Scale [35].
- Depression: Beck Depression Inventory II [22].
- Self-reported physical activity: Physical Activity Scale for the Elderly [36].
- Quality of Life: Parkinson’s Disease Questionnaire 39 [37].
- Self-experienced attention deficits: Self-perceived deficits in attention questionnaire [38].
- Motor impairment: Unified Parkinson’s Disease Rating Scale Part III (UPDRS III) [39].
- Freezing of gait: Freezing of Gait Questionnaire [40].

Parallel test versions were used if available. Neuropsychological assessments were conducted by trained psychologists, neurological tests were carried out by neurologists, physicians in neurological training, or PD nurses.

### Statistical analysis

Data analyses were carried out using SPSS Statistics for Windows, Version 25.0 (Armonk, NY: IBM Corp). To investigate long-term effects of the CT group in comparison to PT, 3 × 2 (time × group) mixed repeated measures analyses of variances (ANOVA) were computed for primary and secondary outcome variables. An effect was considered significant at *p *≤ 0.05. As we used two primary outcome scores, we used Bonferroni correction for multiple testing and therefore considered an effect as significant at *p* ≤ 0.025. Due to the exploratory character, no alpha-correction was applied for the secondary outcome analyses. Partial eta square (*η*^2^) is reported as effect size, indicating small effects from *η*^2^ = 0.01 to *η*^2^ ≤ 0.06, medium effects from *η*^2^ > 0.06 to *η*^2^ < 0.14, and large effects from *η*^2^ ≥ 0.14 [41]. To avoid the risk of drop-out associated bias, we report the results of a per-protocol (PP) approach as well as of an intention-to-treat (ITT) approach for the ANOVAs. For the PP approach, only patients who completed the respective follow-up assessment were included in the analyses; for the ITT approach, missing data were imputed using the Last Observation Carried Forward (LOCF) method.

In case of a significant time x group interaction effect, test-specific post-hoc analyses were calculated to examine direction and temporal course of the effect. For this purpose, change scores were computed by subtracting baseline scores from 6 and 12 months follow-up scores, and tested for normal distribution using the Shapiro–Wilk test. Afterwards, change score differences between the intervention groups were compared with independent samples *t *tests or Mann–Whitney *U* tests, respectively. Moreover, paired *t *tests for dependent samples, respectively, Wilcoxon tests were computed to detect significant mean score changes over time within both groups. Post-hoc significance levels were Bonferroni corrected for the number of cognitive tests within the respective domain.

Furthermore, we examined possible predictors of intervention responsiveness. Intervention responsiveness was operationalized by the change scores (differences between baseline level of the respective cognitive outcome score and the performance at follow-up assessment). Therefore, multiple linear regression analyses were performed for the 6 months as well as for the 12 months change scores. Concerning the training’s specificity, the analyses were computed for both intervention groups. Following studies with healthy older adults and PD-MCI patients [42–49], we included as predictors the baseline level of the respective outcome variable, age, sex, education level, and ApoE4 status. Regarding PD characteristics, we added UPDRS III and levodopa equivalent daily dose (LEDD) as possible predictors what is in line with Kalbe et al. [15].

## Results

### Dropout analysis

Initially, 76 patients were screened for eligibility and after pretest 64 patients were randomly allocated to the CT group (*n *= 33) or PT (*n *= 31), respectively. The dropout rate during the intervention phase was 4.7% (CT: *n *= 2, PT: *n *= 1). Out of the 61 patients who completed the pre- and post-intervention assessments, 54 patients completed the 6 months (CT: *n *= 28, PT: *n *= 26) and 49 patients completed the 12 months follow-up assessment (CT: *n *= 25, PT: *n *= 24). Dropout rates were 11.5% from baseline to 6 months follow-up and 9.3% from 6 to 12 months follow-up. Reasons for dropout were illness other than PD that made further participation impossible (CT: *n *= 2, PT: *n *= 2), loss of contact (CT: *n *= 1, PT: *n *= 3), patients’ wish to stop participation (CT: *n *= 2, PT: *n *= 1), and deep brain stimulation (CT: *n *= 1), see also Supplementary Fig. 1 (online resource). Drop-out patients did not significantly differ from patients who completed the study in terms of age (*p *= 0.281, Mann–Whitney *U* test), sex (*p *= 0.223, Fisher’s exact test), intervention group (*p *= 1.000, Fisher’s exact test), and motor impairment (*p *= 0.409, Mann–Whitney *U* test).

### Comparability between groups

Sociodemographic and clinical baseline characteristics of the subgroups included in the 6 and 12 months follow-up analyses can be seen in Table 1. The intervention groups were comparable with regard to age, sex distribution, education, disease onset, disease duration, severity of motor symptoms, LEDD, ApoE4 state, and depression. Further, we checked for comparability between groups concerning the training participation. Patients included in the 6 months follow-up analysis participated in 11 of the 12 training sessions (median; CT range: 8–12, PT range: 9–11) independent of group affiliation (*χ*^2^ = 5.333; *p *= 0.255). For the 12 months follow-up groups median and range did not change (*χ*^2^ = 2.536; *p *= 0.638).

### Long-term effects of the cognitive training

**Fig. 1:**
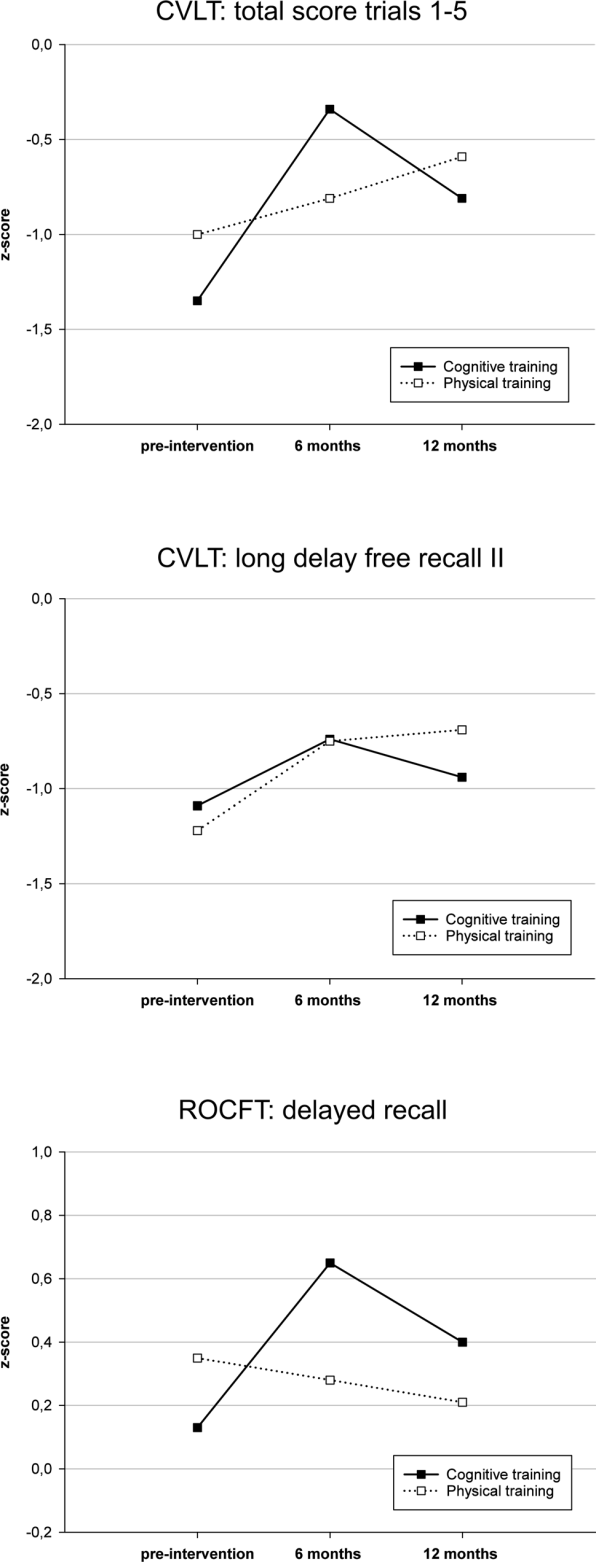
Memory domain *z*-scores pre-intervention and at 6 and 12 months follow-up assessments for both training groups

Table 2 presents the results of the training effects analyses. Regarding the primary outcome variables, time × group interaction was significant for memory composite score (PP: *p *= 0.006, *η*^2^ = 0.214; ITT: *p *= 0.023, *η*^2^ = 0.123), indicating a medium effect size favouring the CT group. Interaction effects for the executive functions composite score as well as for all secondary cognitive and non-cognitive outcomes did not reach significance. Post-hoc tests showed that change scores are significantly higher in the CT group than in the PT group at 6 months follow-up for CVLT total score (*p *= 0.011), and ROCFT delayed recall (*p *= 0.014), however, there were no significant change score differences at 12 months follow-up assessment (Table 3). Moreover, paired *t* tests showed significantly better test results at 6 months follow-up compared to baseline assessment for CVLT total score (*p *< 0.001), and ROCFT delayed recall (*p *= 0.002) in the CT group. No significant differences were found between pre-intervention and 12 months follow-up assessment. In the PT group, there were significant differences between baseline and 6 as well as 12 months follow-up assessments for CVLT delayed recall (*p *= 0.001 respective *p *= 0.013) with better test results at the follow-up assessments. All significant results indicate an improvement over time. Between 6 and 12 months follow-up, there were no significant memory changes in either group. The results are presented in Table 4. Figure 1 illustrates the course of the memory scores in both groups.

**Table 2:** Training effects for both intervention groups

	Cognitive training			Physical activity			PP—rmANOVA		ITT—rmANOVA	
	Pre (*n* = 28)	6 months (*n* = 28)	12 months (*n* = 25)	Pre (*n* = 26)	6 months (*n* = 26)	12 months (*n* = 24)	*p* (time × group)	Partial Eta^2^	*p* (time × group)	Partial Eta^2^
Primary outcomes										
Memory composite score	− 0.72 ± 0.78	− 0.14 ± 0.92	− 0.45 ± 0.81	− 0.62 ± 0.89	− 0.43 ± 0.85	− 0.36 ± 1.12	**0.006**	**0.214**	**0.023**	**0.123**
Executive functions composite score	− 0.03 ± 0.51	− 0.01 ± 0.46	− 0.09 ± 0.50	− 0.01 ± 0.83	− 0.07 ± 0.73	− 0.20 ± 0.84	0.967	0.002	0.916	0.003
Secondary outcomes										
Attention composite score	− 0.79 ± 0.99	− 0.68 ± 0.93	− 0.61 ± 0.89	− 0.93 ± 1.09	− 0.65 ± 0.93	− 0.58 ± 1.02	0.907	0.005	0.766	0.009
Working memory composite score	0.10 ± 0.75	− 0.33 ± 0.96	0.00 ± 0.72	0.10 ± 0.79	− 0.18 ± 0.95	− 0.12 ± 1.20	0.560	0.025	0.376	0.033
Visuocognition composite score	0.06 ± 0.97	0.25 ± 0.89	0.23 ± 0.93	− 0.08 ± 1.15	0.04 ± 1.09	0.10 ± 1.59	0.944	0.003	0.914	0.003
Language composite score	0.16 ± 0.65	0.24 ± 0.62	0.21 ± 0.62	− 0.28 ± 1.14	0.19 ± 0.53	0.08 ± 0.81	0.156	0.079	0.253	0.046
Activities of daily living	2.50 ± 1.73	3.03 ± 2.64	2.46 ± 1.95	2.83 ± 2.07	2.77 ± 2.14	3.26 ± 2.32	0.661	0.026	0.615	0.021
Self-reported physical activity	137.1 ± 77.2	144.2 ± 111.8	134.4 ± 56.8	126.0 ± 61.1	131.3 ± 76.8	122.0 ± 66.7	0.362	0.048	0.419	0.031
Depression	8.43 ± 5.65	10.75 ± 7.98	11.63 ± 8.96	7.28 ± 4.11	9.74 ± 6.22	9.55 ± 6.27	0.475	0.037	0.943	0.002
Quality of life	35.71 ± 19.59	32.71 ± 25.15	34.32 ± 26.03	28.04 ± 14.31	27.75 ± 13.83	33.29 ± 20.63	0.370	0.049	0.553	0.022
Self-experienced deficits of attention	100.92 ± 15.08	101.35 ± 19.15	102.23 ± 17.89	102.44 ± 16.09	106.04 ± 14.43	100.12 ± 17.85	0.112	0.087	0.183	0.068
UPDRS III	25.43 ± 13.26	22.67 ± 7.37	26.80 ± 10.53	25.08 ± 12.80	24.04 ± 8.60	25.79 ± 12.80	0.684	0.017	0.260	0.045
FOG	8.33 ± 6.52	8.85 ± 5.76	8.60 ± 6.21	5.42 ± 4.07	5.83 ± 4.28	6.50 ± 5.09	0.316	0.055	0.476	0.026

Data are given in mean ± standard deviation; significant results after Bonferroni correction are in bold

*FOG* Freezing of Gait, *ITT* intention-to-treat analysis, *PP* per-protocol analysis, *rmANOVA* repeated measures analysis of variance, *UPDRS* Unified Parkinson’s Disease Rating Scale

**Table 3:** 6 and 12 months memory change score differences between cognitive training and physical activity group

	6 months change score		*t* test		12 months change score		*t* test	
	Cognitive training	Physical activity	*T*	*p*	Cognitive training	Physical activity	*T*	*p*
CVLT total score trials 1–5	1.01 ± 1.02	0.24 ± 0.94	** − 2.541**^**a**^	**0.011**^**a**^	0.51 ± 1.31	0.46 ± 1.56	− 0.460^a^	0.645^a^
CVLT long delay free recall II	0.41 ± 0.85	0.53 ± 0.58	− 0.616	0.540	0.19 ± 1.07	0.53 ± 0.97	− 1.170	0.248
ROCFT delayed recall	0.57 ± 0.84	− 0.03 ± 0.84	**2.540**	**0.014**	0.32 ± 0.75	− 0.07 ± 1.05	1.488	0.144

6 months change scores are defined as Δ 6 months follow-up—pre-intervention *z*-scores; 12 months change scores are defined as Δ 12 months follow-up—pretest *z*-scores; data are given in mean ± standard deviation, significant results after Bonferroni correction are in bold

*CVLT* California Verbal Learning Test, *ROCFT* Rey-Osterrieth Complex Figure Test

^a^Mann—Whitney *U* tests were used

**Table 4:** Memory test results before intervention and at 6 and 12 months follow-up assessment in both intervention groups

	Pre-intervention	6 months	12 months	pre-intervention vs. 6 months		pre-intervention vs. 12 months		6 months vs. 12 months	
				*T/Z*	*p*	*T/Z*	*p*	*T/Z*	*p*
Cognitive training	*n* = 28	*n* = 28	*n* = 26						
CVLT total score trials 1–5	− 1.35 ± 1.35	− 0.34 ± 1.33	− 0.81 ± 1.19	** − 5.223**^**a**^	** < 0.001**^**a**^	− 1.964^a^	0.061^a^	2.111^a^	0.045 ^a^
CVLT long delay free recall II	− 1.09 ± 1.04	− 0.74 ± 1.17	− 0.94 ± 1.09	− 2.486^a^	0.020^a^	0.868^a^	0.395^a^	1.594^a^	0.124 ^a^
ROCFT delayed recall	0.13 ± 0.93	0.65 ± 0.96	0.40 ± 0.71	** − 3.482**^**a**^	**0.002**^**a**^	− 2.088^a^	0.048^a^	− 1.588^b^	0.112^b^
Physical activity	*n* = 26	*n* = 26	*n* = 24						
CVLT total score trials 1–5	− 1.00 ± 1.20	− 0.81 ± 1.30	− 0.59 ± 1.66	− 1.261^a^	0.219^a^	− 1.130^b^	0.259^b^	− 0.373^b^	0.709^b^
CVLT long delay free recall II	− 1.22 ± 1.00	− 0.75 ± 1.10	− 0.69 ± 1.44	** − 3.477**^**b**^	**0.001**^**b**^	** − 2.693**^**a**^	**0.013**^**a**^	− 0.308^b^	0.758^b^
ROCFT delayed recall	0.35 ± 1.16	0.28 ± 0.89	0.21 ± 0.78	0.173^a^	0.864^a^	0.343^a^	0.735^a^	− 1.049^b^	0.294^b^

Data are given in mean ± standard deviation; significant results after Bonferroni correction are in bold

*CVLT* California Verbal Learning Test, *ROCFT* Rey-Osterrieth Complex Figure Test

^a^dependent *t* test for paired samples

^b^Wilcoxon test

### Prediction of long-term effects

Significant models for predicting change scores of the CT group were found within the executive function, visuocognition and language domains as well as for QoL and motor function at 6 months follow-up (0.374 ≤ *R*^2^_adj_ ≤ 0.713). There was no significant regression model for the prediction of training responsiveness in the memory domain after 6 months. At 12 months follow-up, significant predictive models were found within the memory, executive functions, attention, working memory, visuocognition, and language domains as well as for self-reported physical activity and QoL (0.337 ≤ *R*^2^_adj_ ≤ 0.651). A lower baseline level in the respective outcome variable significantly predicted training gains in almost all significant regression models, the only exceptions were the QoL models. Additionally, higher respective lower age, female respective male sex, higher education level, lower baseline motor status and LEDD, and positive respective negative ApoE4 status were significant predictors for training gains in some secondary outcome parameters after CT. For the PT group, significant regression models were found for the prediction of memory, executive, visuocognitive, language, motor function and ADL change scores after 6 or 12 months (0.374 ≤ *R*^2^_adj_ ≤ 0.961) with lower baseline levels as significant predictors for training responsiveness in all cases, and higher age, male sex, higher education level, lower baseline UPDRS III score, and higher baseline LEDD as significant predictors in few single variables. All significant regression models are presented in Supplementary Tables 1 and 2 (online resource).

## Discussion

We report the long-term results of a multicenter RCT assessing the effects of CT in comparison to an active control training in PD-MCI. In our previous report [15], we could show that CT is feasible and safe for PD patients. Furthermore, we provided evidence for an enhancement of executive functions shortly after CT compared to PT. In the present study, we extended these results by demonstrating training gains of the CT group in the memory domain after 6 months. The main results for 6 and 12 months follow-up assessments were: (i) CT enhanced memory functions after 6 months while there was no positive effect after 12 months, (ii) there were no significant improvements of executive functions or other cognitive and non-cognitive parameters at 6 and 12 months follow-up assessments, (iii) training gains in the memory domain cannot be predicted by means of baseline score, age, sex, education, LEDD, or ApoE4 state. These results provide Class 1 evidence for memory enhancement following CT after 6 months given the multi-center randomized and single-blinded design.

We found a significant interaction effect for the memory composite score indicating an enhancement of memory performance in the CT group. This effect remained after imputing missing data. Post-hoc analyses showed that the significant interaction effect is driven by significant verbal and nonverbal memory improvement of the CT group from baseline to 6 months follow-up assessment while after 12 months the test performance declines. The largest CT improvement was demonstrated for the CVLT total score trials 1–5, a marker for the multidimensional construct of verbal learning. Remarkably, a comparable word list learning score turned out to be the most sensitive memory score for detecting memory dysfunction and cognitive impairment in PD-MCI patients [50], indicating that CT is enhancing highly vulnerable memory functions. Memory functions as primary outcome were expected to improve as the NEUROvitalis program includes training sessions focusing on the memory domain. Moreover, an enhancement in memory functioning after CT could be shown in previous PD studies [13, 51, 52], however, these studies examined the training effect immediately after intervention. Also Alloni et al. [53] demonstrated significant memory improvement immediately after CT while six months after training, the improvement remained for one out of three memory test variables. Notably, in our study, the CT group did not benefit shortly after intervention regarding memory functioning, but only on the 6 months follow-up assessment. This result is consistent with a study from Lawrence et al. [54] who could show a significant verbal memory improvement 12 weeks after CT while immediately after CT this effect did not reach significance. One possible explanation for the delayed effect could be that CT contributes to the development of cognitive strategies what first results in an enhancement of executive functioning (as we found in our study immediately after training, see Kalbe et al. [15]) and is later transferred to memory performance. An argument for this hypothesis is the high strategic load of the CVLT due to the possibility of semantic clustering. Therefore, an influence of executive control on CVLT performance was demonstrated for patients with PD [55], PD dementia [56], mixed neurological patients [57], and older adults with suspected dementia [58]. Moreover, Alexander et al. [59] showed that patients with frontal lesions have difficulties in the CVLT due to poor implementation of a strategy of subjective organization. This explanation may be also applicable to the ROCFT, even though previous studies mainly focused on executive components of the copy condition and few studies provided inconsistent results regarding a strategic load of the recall condition [60, 61]. Test–retest effects must also be considered as an explanation for the delayed memory improvement as at baseline assessment and 6 months follow-up assessment the same test version was used while immediately after intervention and at 12 months follow-up assessment a parallel version was conducted. However, there are two arguments against this suggestion. First, we found a significant time × group interaction effects while a test–retest effect would affect both groups. Second, there are no relevant mean *z*-score differences between post-intervention assessment (results reported by Kalbe et al. [15]) and 12 months follow-up for CVLT total score (CT: *p *= 0.638, PT: *p *= 0.148) and ROCFT delayed recall (CT: *p *= 0.271, PT: *p *= 0.957) in either group, although the same test version was used in these assessments.

Regarding executive functions, the pre-post analyses showed a significant enhancement immediately after the training in the CT group compared to the PT group [15], however, after 6 and 12 months these results did not longer remain evident. Similar results for PD patients were found in the studies from Lawrence et al. [54] and Alloni et al. [53] in which training effects in executive functioning were significant immediately after CT, but mostly not at follow-up assessment (12 and 24 weeks, respectively). Similarly, in MCI patients without PD it has been demonstrated that CT impact is strong in the short-term, but not always strong enough to maintain efficient functioning in the long-term [62]. Especially with regard to the training effort (for both patients and clinical personal), future studies must examine how training effects can be preserved in the long-term. One possible method may be the conduction of further training sessions periodically after the main intervention (so-called “booster training”) for refreshing the strategies learned. Also, continuous home exercises could prevent from a detraining effect over time.

The regression analyses did not reveal a significant model for predicting memory improvement after 6 months, although memory was the only domain in which significant improvements of the CT group could be demonstrated. Therefore, memory enhancement after CT could not be predicted by means of baseline score, age, sex, education level, motor status (UPDRS III), LEDD, or ApoE4 state, indicating that CT was comparably effective in all patients regardless of specific sociodemographic or disease-related characteristics. For executive functioning and the cognitive and non-cognitive secondary outcome variables, the respective baseline level turned out as main predictor for training gain in almost all cases, more precisely, lower baseline levels were predictive for CT responsiveness in the respective domain. This is in line with the short-term results of our study as lower baseline cognitive levels turned out to be the main predictor for training improvement directly after intervention [15]. Additional, higher respective lower age, female respective male sex, higher education level, lower baseline motor status, lower baseline LEDD, and positive respective negative ApoE4 status predicted training gains after 6 or 12 months in the CT group for selected outcomes. Previous PD studies detected lower baseline scores [12, 14], higher global cognitive status [11], higher fluid intelligence and higher self-efficacy expectancy [14], MCI diagnosis [13], higher educational level [11, 14], longer [10] or shorter disease duration [11], younger age [14], and younger age at PD diagnosis [10] as predictive for enhancements in cognitive functions immediately or 3 months after CT. These inconsistent results may be explained by study-specific differences (e.g., sample size and heterogeneity, cognitive tests used), but may also indicate the challenge of predicting CT responsiveness in cognitively impaired PD patients. In our study, the prediction results of the CT group were comparable to those of the PT group as in both groups a lower cognitive baseline level turned out as the main predictor for training responsiveness after 6 and 12 months. Therefore, a low specificity of the predictions for the type of interventional training is assumed. While the randomization procedure minimized the risk of a regression-to-the-mean effect [63], the predictive character of baseline level in both intervention groups may be explained by unspecific test–retest effects. In conclusion, CT can be recommended in PD-MCI patients irrespective of cognitive, educational or motor level, sex, medication characteristics, and ApoE4 status.

There are a few limitations to our study. First, due to recruitment difficulties, the a priori calculated sample size to achieve 80% power for detecting medium effect sizes was missed. However, as we found significant results, the risk of an underpowered study not being able to detect significant effects was not realized in our study. Second, the persons who carried out the diagnostic assessments were blinded regarding the intervention type, but the blinding was not complete as some patients reported details of intervention despite appropriate instructions. However, blinding is a general challenge in non-pharmacological studies. Third, the study did not include a passive control group what may restrict the clinical relevance as a physical activity training does not reflect clinical routine. However, the active control group is also a strength of our study because the significant effects cannot be attributed to unspecific effects due to the attention which is given to the patients during the training sessions. Nevertheless, future studies with an active and a passive control group should be carried out. Another strength of our study is that it is one of the first RCTs examining long-term effects of CT and its predictors for long-term responsiveness in PD-MCI.

## Conclusions

In summary, this study provides Class 1 evidence that multi-domain group CT enhances memory functions (but not executive functions) in PD-MCI patients in the long-term. The previously reported results of improvements in executive functioning immediately after CT could be extended by a delayed verbal and nonverbal memory improvement 6 months after intervention. Therefore, CT is an effective treatment of memory and executive functions in PD-MCI. No significant predictors could be detected for memory training gain indicating that CT is useful for PD patients unrelated to sociodemographic or disease-related characteristics.

## Supplementary Information

Below is the link to the electronic supplementary material.

Supplementary file1 (PDF 97 KB)

Supplementary file2 (DOCX 38 KB)
