# Supplementary figures and images for: Memory enhancement by multidomain group cognitive training in patients with Parkinson’s disease and mild cognitive impairment: long-term effects of a multicenter randomized controlled trial
Source: J Neurol. 2021 Apr 27;268(12):4655–66. doi: 10.1007/s00415-021-10568-9 (PMC8563628; doi:10.1007/s00415-021-10568-9)

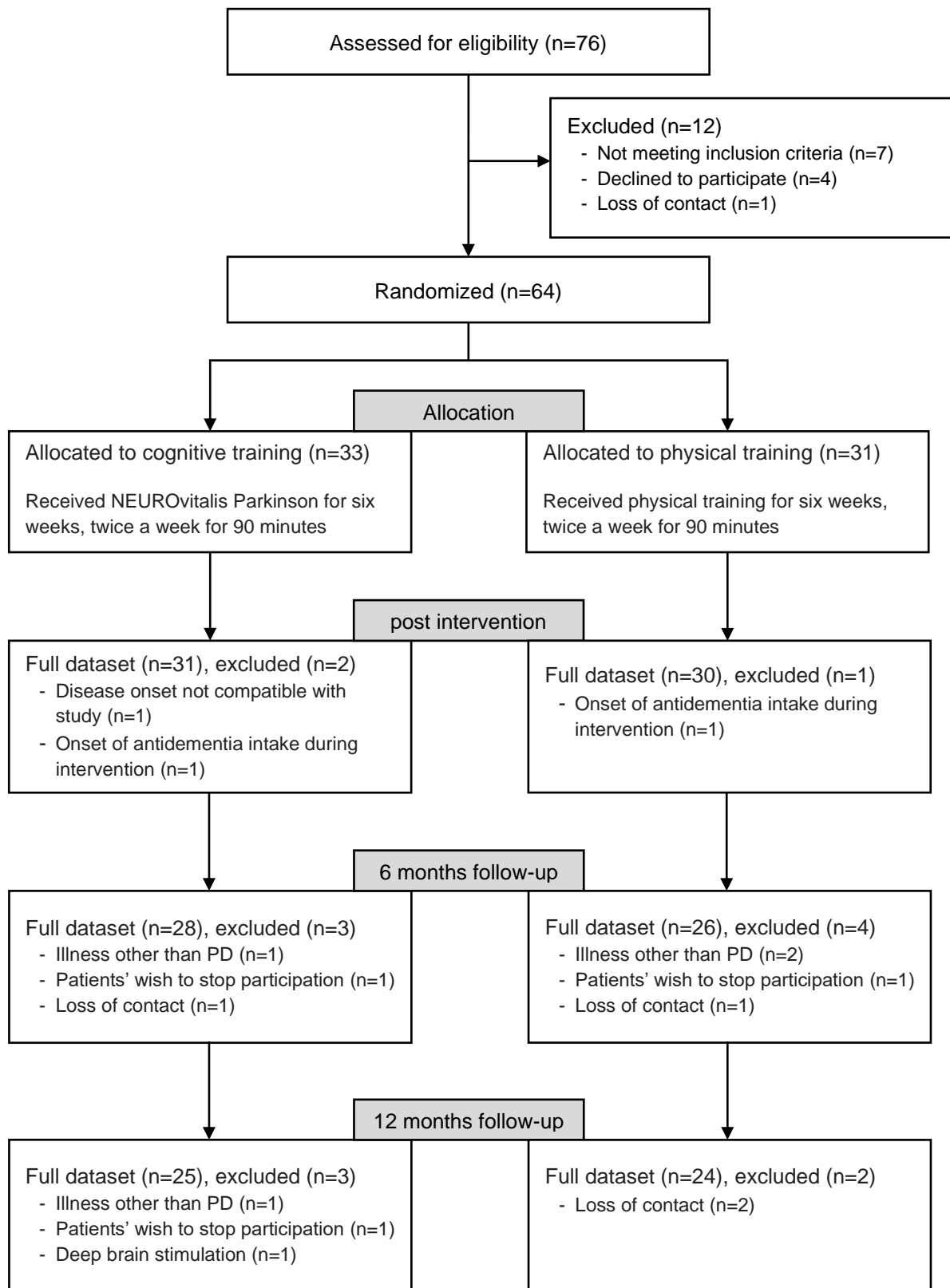

**Supplementary Figure 1.** Participant flow.

Supplement: Supplementary file 1 — Supplementary file1 (PDF 97 KB) [file 415_2021_10568_MOESM1_ESM.pdf]
