# Supplementary material for: Memory enhancement by multidomain group cognitive training in patients with Parkinson’s disease and mild cognitive impairment: long-term effects of a multicenter randomized controlled trial
Source: J Neurol. 2021 Apr 27;268(12):4655–66. doi: 10.1007/s00415-021-10568-9 (PMC8563628; doi:10.1007/s00415-021-10568-9)
Supplement: Supplementary file 2 — Supplementary file2 (DOCX 38 KB) [file 415_2021_10568_MOESM2_ESM.docx]

**Supplementary Table 1.** Regression analyses: predictors for intervention responsiveness after 6 months.

|  |  |  |  | **Standardized coefficients (β) of significant predictors** | | | | | | | | |
| --- | --- | --- | --- | --- | --- | --- | --- | --- | --- | --- | --- | --- |
| **Model** | **F-Test** | **R² (adjusted R²)** | | | Baseline level | Age at baseline | Sex | Education in years | UPDRS III at baseline | LEDD at baseline | ApoE4 status |  |
| **COGNITIVE TRAINING** |  |  | | |  |  |  |  |  |  |  |  |
| **EXECUTIVE FUNCTIONS** |  |  | | |  |  |  |  |  |  |  |  |
| Executive composite score | F(7,16) = 3.029; p = 0.031 | 0.570 (0.382) | | | -0.766*** |  |  |  |  |  |  |  |
| Phonemic word fluency | F(7,19) = 4.166; p = 0.006 | 0.606 (0.460) | | | -0.841*** | 0.471* |  |  |  |  |  |  |
| MCST categories | F(7,16) = 2.960; p = 0.034 | 0.564 (0.374) | | | -0.670** |  |  |  |  |  |  |  |
| **VISUOCOGNITION** |  |  | | |  |  |  |  |  |  |  |  |
| Visuocognition composite score | F(7,19) = 3.458; p = 0.015 | 0.560 (0.398) | | | -0.537* |  | -0.500* |  |  |  |  |  |
| Benton Judgement of Line Orientation | F(7,19) = 3.462; p = 0.015 | 0.561 (0.399) | | | -0.621** |  |  |  |  |  |  |  |
| **LANGUAGE** |  |  | | |  |  |  |  |  |  |  |  |
| ACL | F(7,19) = 9.657; p = 0.000 | 0.781 (0.700) | | | -0.839*** |  |  |  |  |  |  |  |
| **NON-COGNITIVE OUTCOMES** |  |  | | |  |  |  |  |  |  |  |  |
| PDQ-39 | F(7,16) = 3.357; p = 0.021 | 0.595 (0.418) | | |  |  | -0.615* |  | -0.654** |  | 0.522* |  |
| UPDRS III | F(6,19) = 11.343 p = 0.000 | 0.782 (0.713) | | | -0.729*** |  |  |  | --- |  |  |  |
| **CONTROL GROUP** |  |  | | |  |  |  |  |  |  |  |  |
| **EXECUTIVE FUNCTIONS** |  |  | | |  |  |  |  |  |  |  |  |
| Semantic word fluency | F(7,14) = 3.569; p = 0.020 | 0.641 (0.461) | | | -0.729*** |  |  |  |  |  |  |  |
| Phonemic word fluency | F(7,14) = 4.003; p = 0.013 | 0.669 (0.503) | | | -0.657*** |  |  |  |  |  |  |  |
| **VISUOCOGNITION** |  |  | | |  |  |  |  |  |  |  |  |
| Visuocognition composite score | F(7,14) = 2.835; p = 0.046 | 0.586 (0.380) | | | -0.600** |  | 0.530* |  |  |  |  |  |
| ROCFT figure copy | F(7,14) = 3.439; p = 0.023 | 0.632 (0.448) | | | -0.630** |  |  |  |  |  |  |  |
| Benton Judgement of Line Orientation | F(7,14) = 2.945; p = 0.041 | 0.596 (0.393) | | | -0.746** |  |  |  | -0.532* |  |  |  |
| **LANGUAGE** |  |  | | |  |  |  |  |  |  |  |  |
| Language composite score | F(7,14) = 10.543; p = 0.000 | 0.841 (0.761) | | | -0.937*** |  |  |  |  |  |  |  |
| Boston Naming Test | F(7,14) = 6.800; p = 0.001 | 0.773 (0.659) | | | -0.672*** |  |  |  |  |  |  |  |
| ACL | F(7,14) = 75.371; p = 0.000 | 0.974 (0.961) | | | -0.977*** |  |  |  |  |  |  |  |
| **NON-COGNITIVE OUTCOMES** |  |  | | |  |  |  |  |  |  |  |  |
| Bayer ADL | F(7,11) = 6.123; p = 0.004 | 0.796 (0.666) | | | -0.477** |  |  | 0.702** |  | 0.437* |  |  |
| UPDRS III | F(6,14) = 3.346 p = 0.029 | 0.589 (0.413) | | | -0.809*** |  |  |  | --- |  |  |  |

Dependent variables are defined as Δ 6 months follow up - pretest; regression models were computed for composite scores and specific test variables, but only those models that reached statistical significance at p≤0*.*05 are presented; for each significant regression model, only standardized regression coefficients for predictors that have reached statistical significance are reported; negative β coefficients indicate that intervention responsiveness is predicted by lower predictor level, female sex, or negative ApoE4 status, respectively; *p≤0.05; **p≤0.01; ***p≤0.001.

ACL, Aphasia Check List; ADL, activities of daily living; ApoE, apolipoprotein E; CVLT, California Verbal Learning Test; LEDD, levodopa equivalent daily dose; MCST, Modified Wisconsin Card Sorting Test; PDQ-39, Parkinson's Disease Questionnaire; ROCFT, Rey-Osterrieth Complex Figure Test; UPDRS, Unified Parkinson’s Disease Rating Scale.

**Supplementary Table 2.** Regression analyses: predictors for intervention responsiveness after 12 months.

|  |  | |  | | |  | | **Standardized coefficients (β) of significant predictors** | | | | | | |
| --- | --- | --- | --- | --- | --- | --- | --- | --- | --- | --- | --- | --- | --- | --- |
| **Model** | | **F-Test** | | **R² (adjusted R²)** | Baseline level | | Age at baseline | | Sex | Education in years | UPDRS III at baseline | LEDD at baseline | ApoE4 status |  |
| **COGNITIVE TRAINING** | |  | |  |  | |  | |  |  |  |  |  |  |
| **MEMORY** | |  | |  |  | |  | |  |  |  |  |  |  |
| CVLT total score trials 1-5 | | F(7,16) = 3.276; p = 0.023 | | 0.589 (0.409) | -0.726** | |  | | -0.595* |  |  |  |  |  |
| ROCFT delayed recall | | F(7,15) = 3.991; p = 0.012 | | 0.651 (0.488) | -0.679*** | |  | |  |  |  | -0.632** |  |  |
| **EXECUTIVE FUNCTIONS** | |  | |  |  | |  | |  |  |  |  |  |  |
| MCST categories | | F(7,15) = 4.638; p = 0.006 | | 0.684 (0.537) | -0.877*** | |  | |  |  |  |  |  |  |
| Key Search Test | | F(7,16) = 4.589; p = 0.006 | | 0.668 (0.522) | -0.825** | | -0.414* | | 0.733** |  |  | -0.725** | -0.393* |  |
| **ATTENTION** | |  | |  |  | |  | |  |  |  |  |  |  |
| Attention composite score | | F(7,15) = 4.218; p = 0.009 | | 0.663 (0.506) | -0.545** | |  | |  | 0.482* |  | -0.477* |  |  |
| d2-R errors | | F(7,15) = 4.487; p = 0.007 | | 0.677 (0.526) | -0.511* | |  | |  | 0.594* |  | -0.577** |  |  |
| d2-R concentration performance | | F(7,15) = 3.823; p = 0.014 | | 0.641 (0.473) | -0.742*** | |  | |  |  |  |  |  |  |
| **WORKING MEMORY** | |  | |  |  | |  | |  |  |  |  |  |  |
| Letter-Number Sequencing (WAIS) | | F(7,16) = 3.538; p = 0.017 | | 0.608 (0.436) | -0.506* | |  | | -0.454* |  |  |  |  |  |
| Digit span backward (WAIS) | | F(7,16) = 2.668; p = 0.049 | | 0.539 (0.337) | -0.498* | |  | |  |  |  |  | 0.503* |  |
| **VISUOCOGNITION** | |  | |  |  | |  | |  |  |  |  |  |  |
| Benton Judgement of Line Orientation | | F(7,16) = 2.696; p = 0.048 | | 0.541 (0.340) | -0.616** | |  | |  |  |  |  |  |  |
| **LANGUAGE** | |  | |  |  | |  | |  |  |  |  |  |  |
| ACL | | F(7,16) = 7.138; p = 0.001 | | 0.757 (0.651) | -0.735*** | |  | |  |  |  |  |  |  |
| **NON-COGNITIVE OUTCOMES** | |  | |  |  | |  | |  |  |  |  |  |  |
| PASE | | F(7,15) = 6.778; p = 0.001 | | 0.760 (0.648) | -0.756*** | |  | |  |  |  |  |  |  |
| BDI II | | F(7,15) = 3.149; p = 0.030 | | 0.595 (0.406) |  | |  | | -0.462* |  |  |  |  |  |
| PDQ-39 | | F(7,13) = 2.920; p = 0.045 | | 0.611 (0.402) |  | |  | | -0.674** |  |  |  |  |  |
| **CONTROL GROUP** | |  | |  |  | |  | |  |  |  |  |  |  |
| **MEMORY** | |  | |  |  | |  | |  |  |  |  |  |  |
| ROCFT delayed recall | | F(7,14) = 4.538; p = 0.008 | | 0.694 (0.541) | -0.628** | |  | |  |  |  |  |  |  |
| **EXECUTIVE FUNCTIONS** | |  | |  |  | |  | |  |  |  |  |  |  |
| Phonemic word fluency | | F(7,14) = 3.814; p = 0.016 | | 0.656 (0.484) | -0.473* | | 0.716* | |  |  |  | 0.501* |  |  |
| **LANGUAGE** | |  | |  |  | |  | |  |  |  |  |  |  |
| Language composite score | | F(7,14) = 2.796; p = 0.048 | | 0.583 (0.374) | -0.741** | |  | |  |  |  |  |  |  |
| ACL | | F(7,14) = 19.074; p = 0.000 | | 0.905 (0.858) | -0.911*** | |  | |  |  |  |  |  |  |
| **NON-COGNITIVE OUTCOMES** | |  | |  |  | |  | |  |  |  |  |  |  |
| Bayer ADL | | F(7,10) = 4.283; p = 0.019 | | 0.750 (0.575) | -0.764*** | |  | |  |  |  |  |  |  |

Dependent variables are defined as Δ 12 months follow up - pretest; regression models were computed for composite scores and specific test variables, but only those models that reached statistical significance at p≤0*.*05 are presented; for each significant regression model, only standardized regression coefficients for predictors that have reached statistical significance are reported; negative β coefficients indicate that intervention responsiveness is predicted by lower predictor level, female sex, or negative ApoE4 status, respectively; *p≤0.05; **p≤0.01; ***p≤0.001.

ACL, Aphasia Check List; ADL, activities of daily living; ApoE, apolipoprotein E; BDI, Beck Depression Inventory; CVLT, California Verbal Learning Test; LEDD, levodopa equivalent daily dose; MCST, Modified Wisconsin Card Sorting Test; PASE, Physical Activity Scale for the Elderly; PDQ-39, Parkinson's Disease Questionnaire; ROCFT, Rey-Osterrieth Complex Figure Test; UPDRS, Unified Parkinson’s Disease Rating Scale; WAIS, Wechsler Adult Intelligence Scale.
